# The Impact of Ultra-Processed Foods on Chronic Kidney Disease Progression

**DOI:** 10.3390/nu18183055

**Published:** 2026-09-18

**Authors:** Ewelina Młynarska, Katarzyna Hossa, Agata Kłosowicz, Natalia Krupińska, Przemysław Kuzar, Adrian Majtas, Zofia Możdżan, Aleksandra Prusak, Patrycja Szymańska, Anna Wieczorek, Jacek Rysz, Beata Franczyk

**Affiliations:** 1 Department of Nephrocardiology, Medical University of Lodz, 90-419 Lodz, Polandadrian.majtas@stud.umed.lodz.pl (A.M.);; 2 Department of Nephrology, Hypertension and Internal Medicine, Medical University of Lodz, 90-549 Lodz, Poland

**Keywords:** kidney chronic disease, ultra-processed foods, nutrition

## Abstract

Ultra-processed foods (UPFs) constitute an increasing proportion of modern diets and may contribute to the development and progression of chronic kidney disease (CKD). This review summarizes current evidence linking UPF consumption with adverse kidney outcomes and discusses the underlying pathophysiological mechanisms. UPFs are typically high in sodium, added sugars, unhealthy fats and food additives, while being low in dietary fiber and essential micronutrients. Their consumption may promote inflammation and oxidative stress, metabolic acidosis, hypertension, gut microbiota dysbiosis, insulin resistance, and metabolic syndrome. Epidemiological studies consistently associate higher UPF intake with an increased risk of incident CKD, an accelerated decline in the estimated glomerular filtration rate, CKD progression and unfavorable cardiovascular and mortality outcomes. Reducing UPF intake and replacing these products with minimally processed plant-based foods may therefore represent a practical dietary strategy for kidney protection. However, most available evidence is observational, and further prospective and interventional studies are required to establish causality and define CKD-specific dietary recommendations.

## 1. Introduction

Chronic kidney disease (CKD) is defined as abnormalities of kidney structure or function, such as a reduced estimated glomerular filtration rate (eGFR < 60 mL/min/1.73 m^2^) or an increased urinary albumin-to-creatinine ratio (ACR > 3 mg/mmol), that persist for at least 3 months and have health implications [1,2]. According to the World Health Organization (WHO), approximately 674 million people worldwide have CKD, with the majority living in low- and middle-income countries [3]. CKD is associated with progression to end-stage kidney disease (ESKD), cardiovascular complications, and premature mortality [4]. It is the ninth leading cause of death globally, accounting for approximately 1.5 million deaths annually [5].

The development and progression of CKD are influenced by multiple risk factors, with diabetes mellitus and hypertension remaining the leading ones [6]. Management of modifiable risk factors, including maintaining a healthy body weight, optimizing blood pressure control, reducing salt intake, and adopting a healthy lifestyle, may help prevent CKD and delay its progression [6]. Among these factors, diet represents a potentially modifiable determinant of kidney health, as both dietary patterns and food processing practices may influence CKD development and progression [7].

Foods can be classified according to the NOVA classification system, which categorizes food products into four groups based on the nature, extent, and purpose of the industrial processing they undergo before consumption [8]. According to this classification, ultra-processed foods (UPFs) are industrial formulations produced from extensively modified food components and additives through multiple complex processes. They typically contain ingredients rarely used in culinary preparations and are designed to be convenient, highly palatable, and shelf-stable products that may displace minimally processed foods and freshly prepared meals [8]. UPF consumption varies substantially between countries, ranging from approximately 10–20% of total energy intake in Mediterranean countries to 45–60% in countries such as Canada, Australia, the United Kingdom, and Sweden [9,10]. Approximately 70% of the United States food supply consists of foods generally classified as ultra-processed [11].

**Figure 1:**
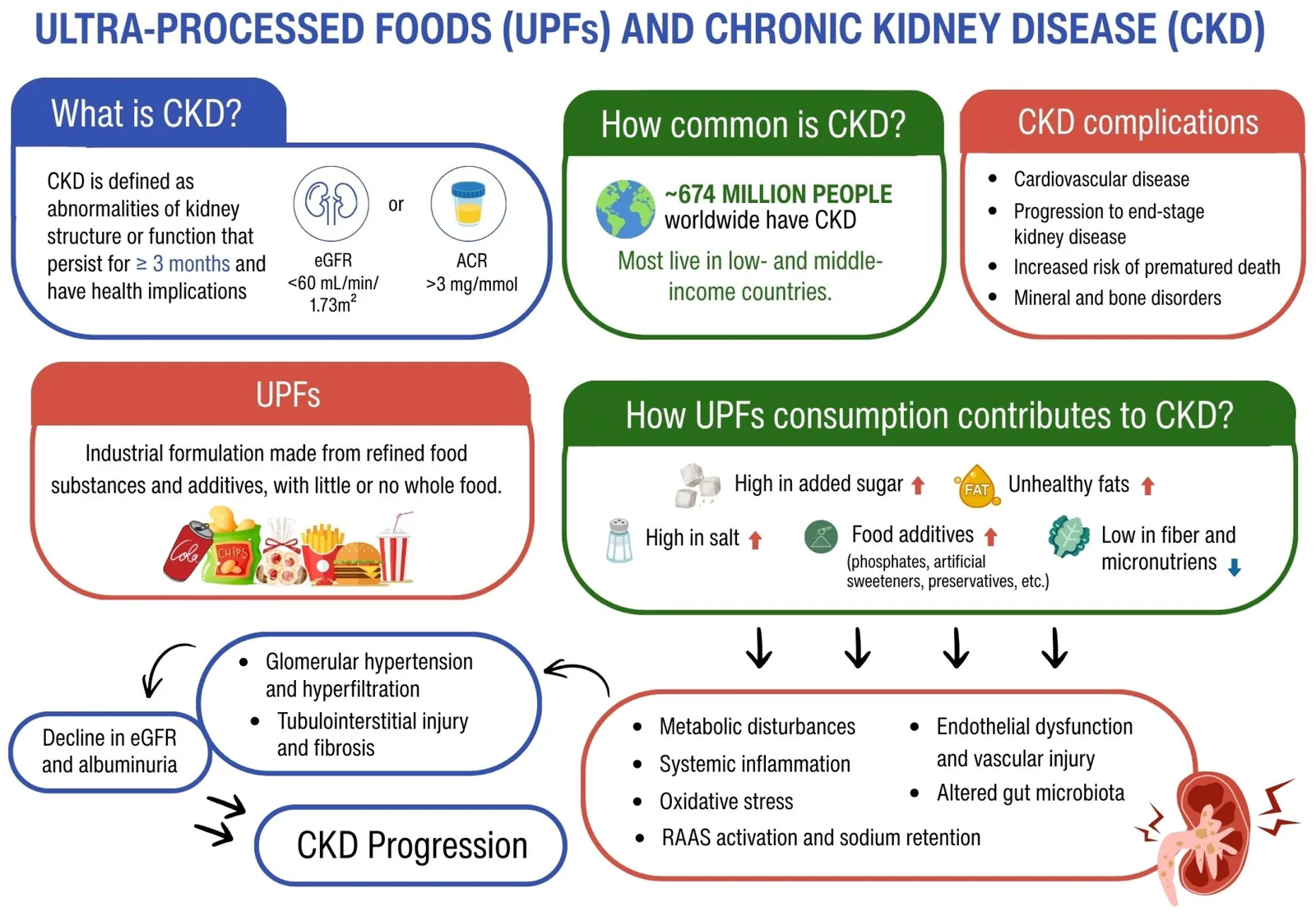
The link between UPF consumption and the development and progression of CKD [1,3,4,7,8,12,13,14].

High consumption of UPFs has been associated with an increased risk of several chronic diseases, including CKD. Moreover, UPF intake has been linked to a decline in eGFR [12]. These associations may be particularly relevant in patients with established CKD, as a high UPF intake may further aggravate metabolic disturbances that occur with declining kidney function [13]. UPFs often contain high amounts of added sugars, salt, unhealthy fats, and mineral-containing additives, which may contribute to metabolic disturbances relevant to CKD progression [14]. Figure 1 illustrates the link between UPF consumption and the development and progression of CKD.

This review summarizes current evidence on the association between ultra-processed food consumption and chronic kidney disease progression, with a focus on potential mechanisms, kidney-related outcomes, and clinical implications.

## 2. Methods: Literature Search and Article Selection

This narrative review was developed through a focused literature search of PubMed, Scopus, and Google Scholar. The search included publications addressing ultra-processed food consumption, the NOVA classification, gut microbiota, intestinal permeability, systemic metabolic disturbances, inflammation, oxidative stress, dietary acid load, uremic toxins, and chronic kidney disease. Combinations of the following terms were used: “ultra-processed food”, “processed food”, “NOVA classification”, “gut microbiota”, “inflammation”, “oxidative stress”, “metabolic acidosis”, “uremic toxins”, “kidney disease”, “chronic kidney disease”, “eGFR”, and “albuminuria”.

Articles were selected based on their relevance to the objectives of this review, with priority given to systematic reviews, meta-analyses, prospective cohort studies, mechanistic studies, clinical studies, and relevant guideline or consensus publications. Studies were excluded when they were unrelated to UPF exposure or kidney outcomes, lacked sufficient methodological information, or were duplicate publications. Reference lists of key articles were also screened to identify additional relevant studies. Because this was a narrative review, the search and selection process was intended to identify and synthesize the most relevant evidence rather than to provide a fully systematic assessment of all available publications.

## 3. Definition and Characteristics of Ultra-Processed Foods

### 3.1. NOVA Classification System

The NOVA classification system was first proposed by Monteiro and colleagues at the University of São Paulo in 2009 to categorize foods according to the nature, extent, and purpose of industrial processing rather than their nutritional composition. It was developed to better reflect the role of food processing in determining diet quality and its relationship with health and disease. Since its introduction, the NOVA system has been increasingly applied in nutritional epidemiology and public health research across numerous countries [15].

Within the NOVA classification system, foods are categorized into four groups according to the extent and purpose of industrial processing. Group 1 comprises unprocessed or minimally processed foods, while Group 2 includes processed culinary ingredients, such as oils, fats, sugar, and salt, which are obtained from Group 1 foods or directly from natural sources and are primarily used in meal preparation. Group 3 consists of processed foods produced by combining Group 1 foods with Group 2 ingredients using preservation methods such as canning or fermentation. Group 4 comprises UPFs, which are manufactured primarily from food-derived substances, ingredients of exclusive industrial use, and additives, with little or no intact whole foods remaining. These products are formulated to provide convenience, enhanced palatability, and prolonged shelf life [16]. The NOVA food classification system is presented in Table 1.

Although the NOVA classification includes four food groups, scientific research has focused primarily on Group 4, which comprises UPFs. This interest is driven by the substantial increase in UPF consumption worldwide and the growing body of evidence linking higher UPF intake with adverse health outcomes. Consequently, UPFs have become a major focus of epidemiological and clinical research investigating the relationship between industrial food processing and chronic diseases, including CKD [12].

### 3.2. Nutritional Profile of Ultra-Processed Foods

UPFs are characterized by a distinct nutritional profile resulting from industrial formulation and extensive processing. Compared with minimally processed foods, they typically have a higher energy density and lower nutritional quality, reflecting both the ingredients used and the extent of industrial processing [13,21]. Unlike minimally processed foods, UPFs are produced primarily from refined food substances and ingredients intended for industrial use, including refined starches, vegetable oils, protein isolates, and added sugars, with only limited amounts of intact whole foods. Their manufacture involves numerous industrial processes, such as refining, fractionation, extrusion, hydrolysis, hydrogenation, and molding, which improve product stability, convenience, and shelf life [16,21].

Consequently, UPFs generally contain higher levels of sodium, added sugars, and saturated fats, while providing lower amounts of dietary fiber, vitamins, and minerals than minimally processed foods [13,21]. Dietary patterns characterized by a high intake of UPFs have been consistently associated with poorer dietary quality and lower adherence to healthy dietary patterns [13]. Beyond their characteristic nutrient composition, increased consumption of UPFs is associated with the gradual displacement of minimally processed foods from the diet. As the proportion of UPFs in the diet increases, the intake of fruits, vegetables, legumes, and cereals generally declines, resulting in lower intakes of dietary fiber, protein, potassium, magnesium, zinc, and several vitamins, including vitamins A, C, D, E, and B12 [17].

In addition to their characteristic nutritional composition, UPFs are distinguished by the extensive use of food additives, incorporated during industrial processing. These include preservatives, emulsifiers, stabilizers, colorants, flavorings, flavor enhancers, non-nutritive sweeteners, and texturizing agents, which are added to improve product stability, texture, appearance, palatability, and shelf life. The extensive use of these additives, combined with industrial formulations designed to enhance sensory properties and convenience, represents one of the defining characteristics of UPFs and distinguishes them from minimally processed foods [22].

### 3.3. Common Dietary Sources of Ultra-Processed Foods

UPFs comprise a wide range of industrially produced food and beverage products. Major dietary sources include sugar-sweetened beverages, bakery products, breakfast cereals, confectionery, sweet and savory snacks, ready-to-eat meals, processed meat products, fast-food items, flavored dairy products, desserts, and ice cream. Although the contribution of individual food categories differs across populations, these products constitute a substantial share of UPF consumption worldwide [10,16,23].

Despite significant differences in the overall consumption of UPFs across Europe, similar food categories consistently account for the largest share of UPF intake. Fine bakery wares, processed meat products, and composite dishes were identified as the principal contributors to UPF intake, whereas soft drinks were the main ultra-processed beverage in most European countries [9].

The widespread consumption of these products is further driven by their broad availability in retail environments and intensive marketing efforts. Consequently, UPFs have become an increasingly prominent component of people’s daily diets. Furthermore, these products are increasingly replacing freshly prepared meals and minimally processed foods, becoming an integral component of contemporary dietary patterns across different population groups [23].

## 4. Pathophysiological Mechanisms Linking Ultra-Processed Foods to Chronic Kidney Disease Progression

### 4.1. Nutritional Composition: Ultra-Processed Versus Minimally Processed Foods

The impact of UPFs on renal function is based on their nutritional profile when compared to unprocessed or minimally processed foods. According to the NOVA classification system, minimally processed foods provide a balanced ratio of essential dietary fibers, natural antioxidants and electrolytes like potassium, which have nephroprotective effects [16]. In contrast, UPFs involve extensive industrial fracturing that gradually strips away these protective components. To achieve an extended shelf life, UPFs are engineered with supraphysiological concentrations of dietary sodium, refined carbohydrates and sugars, varying from the nutritional composition of minimally processed alternatives [23].

This nutritional divergence acts as a catalyst for the mechanisms of CKD progression. The disproportionately high sodium content in UPFs compared to minimally processed foods drives chronic extracellular volume expansion. In patients with decreased renal excretion, this leads to systemic hypertension and accelerates mechanical microvascular damage [16]. Altogether, it creates an environment where elevated advanced glycation end-products (AGEs) and unchecked ROS trigger persistent inflammation and profound oxidative stress, accelerating the structural degradation of renal tissues [24]. The main pathophysiological mechanisms linking UPF consumption to CKD progression are summarized in Table 2. 

### 4.2. Inflammation and Oxidative Stress

Recent epidemiological evidence links the consumption of UPFs and pro-inflammatory diets to the accelerated onset and progression of CKD. Large-scale meta-analyses and prospective cohort studies establish a clear, dose-dependent relationship between poor dietary patterns and renal decline. Individuals consuming the highest amounts of UPFs face a 25% elevated risk of incident CKD compared to those with the lowest intake, with dose–response data indicating that every 10% increase in the dietary proportion of UPFs yields a 7% higher disease risk [34,35]. Similarly, the application of the Dietary Inflammatory Index (DII) in large cohorts demonstrates that individuals with the most pro-inflammatory diets carry a 34% higher risk of developing CKD.

The pathogenesis of this epidemiological trend is connected to severe metabolic dysregulation and gut dysbiosis caused by the distinct nutritional profile of UPFs. Characterized by high energy density, refined carbohydrates, saturated fats, synthetic additives, and fiber deficit, these diets act as persistent exogenous stressors [13,34]. This increased intestinal permeability facilitates the systemic translocation of pro-inflammatory endotoxins and gut-derived uremic toxins [13,16]. Moreover, the extensive industrial techniques used to manufacture UPFs introduce highly bioavailable inorganic phosphates and advanced glycation end-products (AGEs), which, alongside a deficiency in protective exogenous antioxidants, severely overwhelm the body’s antioxidant defenses [13,34,36].

This failure of antioxidant defense triggers the accumulation of reactive oxygen species (ROS) and the systemic circulation of key inflammatory markers, including *C*-reactive protein (CRP), interleukin-6 (IL-6) and tumor necrosis factor-alpha (TNF-α) [36]. The accumulation of N2,N2-dimethylguanosine, an RNA byproduct and reliable endogenous biomarker for systemic oxidative stress, provides direct metabolic evidence that UPFs cause cellular damage, while elevated levels of mannose and glucose indicate the development of severe insulin resistance and low-grade systemic inflammation [25].

Consequently, the synergistic cascade of ROS and pro-inflammatory cytokines and persistent metabolic dysregulation lead to the pathological remodeling of renal architecture [13,34,36]. Chronic microvascular damage includes endothelial dysfunction, glomerular hyperfiltration, and the acceleration of tubulointerstitial fibrosis [13,25,36]. Over time, these renal injuries manifest clinically as a significantly increased urinary ACR and decline in eGFR, leading to metabolic acidosis, hyperkalemia and hyperphosphatemia, thereby accelerating the overall decline in renal function [13,36].

To capture the vascular consequences of these UPF-induced pathophysiological processes, biomarkers act as a translational bridge. Specifically, the renal resistive index (RRI), a non-invasive Doppler ultrasonographic marker of intrarenal vascular resistance, serves as a valuable clinical tool to assess this damage. As demonstrated by Provenzano et al., an elevated RRI is independently associated with adverse renal and cardiovascular risk profiles in patients with CKD [37]. The chronic inflammation, systemic insulin resistance and structural remodeling directly contribute to increased intrarenal stiffness and vascular resistance. Available imaging biomarkers like the RRI provide a practical method to monitor dietary-induced renal microvascular remodeling, strongly reinforcing the clinical connection between poor nutritional factors and progressive CKD.

### 4.3. Metabolic Acidosis and Acid Load

The clinical progression of CKD is significantly worsened by UPF-heavy Western dietary patterns, which are characterized by a high potential renal acid load (PRAL) driven by an excess of phosphorus and animal proteins, alongside a deficiency in plant precursors like citrate and potassium [13,38]. This high dietary acid load (DAL) exacerbates uremic metabolic disturbances and operates synergistically with insulin resistance, hyperkaliemia, gut dysbiosis, and hyperphosphatemia. Consequently, this creates a “double trouble” paradigm that severely compounds uremic complications [13].

The pathophysiological bridge between UPF consumption and CKD progression is primarily driven by diet-induced acid stress. To maintain acid–base homeostasis against a constant influx of fixed acids, kidneys mount a compensatory response by upregulating glutamine-dependent ammoniagenesis and acid excretion pathways [19,26,39,40]. This initiates renal injury long before overt clinical metabolic acidosis, manifesting initially as “covert” or normo-bicarbonatemic acidosis, wherein patients exhibit tissue acid retention despite maintaining normal serum bicarbonate levels (21–27 mmol/L) [26]. While studies indicate that low urine citrate excretion can serve as an early biomarker for this covert state [41,42,43], the localized compensatory adaptation triggers the intrarenal activation of hormonal mediators, specifically endothelin-1 (ET-1), angiotensin II (Ang II) and aldosterone [38]. The chronic presence of these profibrotic hormones directly stimulates tubulointerstitial inflammation, progressive nephron destruction, and accelerated GFR decline [37]. As systemic acid accumulation persists, the body attempts to buffer unexcreted hydrogen ions by chronically mobilizing alkaline salts from the skeleton, precipitating severe systemic impairments such as renal osteodystrophy [13,38].

Therapeutic strategies prioritize reducing the DAL. The foundational approach involves minimizing highly acidogenic components, such as processed meats and substituting them with plant-based alternatives, giving great results [44]. Adding two to four cups per day of base-producing fruits and vegetables provides sufficient natural base precursors to slow CKD progression, even without entirely eliminating acidogenic foods [45]. Furthermore, targeted dietetic-nutritional interventions offer a powerful alternative to pharmacological therapies. Implementing an alkalizing Mediterranean diet in early-stage CKD and transitioning to a plant-based or vegan low-protein diet in advanced stages safely neutralize PRAL while simultaneously averting the adverse effects associated with pharmacological alkalis [26].

When dietary modifications are insufficient, pharmacological interventions are used. Sodium bicarbonate (NaHCO_3_) remains the first-line oral therapy due to its high efficacy and wide availability [44,46]. While sodium citrate offers an alternative that metabolizes bicarbonate, its clinical application is restricted by an unpleasant taste, higher cost, and the potential to promote gastric aluminum absorption [47]. Beyond traditional alkalis, new drug technologies have introduced oral, non-absorbable polymers designed to selectively bind gastric hydrochloric acid and facilitate its fecal excretion [45]. The investigational polymer Veverimer, for example, successfully increases serum HCO_3_ levels within 24 h of administration [45] and sustains this elevation for up to 52 weeks. Although this metabolic improvement correlates with objective and subjective enhancements in physical function [48,49], it has not yet been demonstrated to actively slow CKD progression [50].

### 4.4. Hypertension and Sodium Overload

Dietary sodium intake represents a primary pathophysiological determinant of blood pressure (BP) regulation [51]. Consequently, minimizing dietary sodium consumption serves as an effective, evidence-based strategy to lower BP [27,52]. The WHO recommends that adults consume less than 2000 mg of sodium per day. This guideline is backed by strong evidence showing that high sodium intake increases BP and cardiovascular disease risk [53]. This outcome stems from the reduced ability of patients with impaired kidney function to excrete an acute or chronic salt load. Because of this physiological limitation, dietary sodium reduction serves as a straightforward, inexpensive, and effective intervention to lower BP, reduce cardiovascular disease (CVD) complications, and halt progressive renal decline [54].

Processing additives independently contribute to this acid load and lower serum bicarbonate levels. In fact, these processing additives can account for 50% to 100% of the total dietary acid load, directly accelerating renal functional decline [28,55].

Chronic high sodium consumption remains consistently associated with an increased risk of CKD development and a progressive decline in eGFR [55,56,57]. Moderate-quality evidence from a meta-analysis by Bach et al. comprising six studies and 43,772 participants confirms that a high dietary sodium intake is a significant risk factor for CKD [55,56,58]. CVD is the leading cause of death in patients with CKD. Remarkably, these patients are 5 to 10 times more likely to die from CVD than to progress to advanced CKD. This high risk is largely driven by pre-existing comorbidities, particularly hypertension and diabetes [18,29,54].

### 4.5. Gut Microbiota Dysbiosis

The human intestinal microbiota is a dense ecosystem composed of trillions of coexisting microorganisms, predominantly bacteria. Far from being passive residents of the digestive tract, this diverse community functions in tandem with the host to preserve epithelial barrier integrity and regulate systemic metabolic health [59]. The intestinal microbiota is responsible for generating essential nutrients, particularly vitamins K and B [60]. It also plays a key role in systemic immune homeostasis by regulating white blood cell responses and defending against pathogenic invasion [61]. Moreover, it modulates human behavior and neurological function via the gut–brain axis [62]. This highlights the fundamental importance of promoting a diverse and resilient gut microbiota through therapeutic dietary interventions and lifestyle changes.

Diet represents the most potent environmental factor impacting gut microbiota composition. Specifically, fiber-rich dietary patterns stimulate the growth of beneficial bacteria, including Bifidobacterium and Lactobacillus, which drive saccharolytic fermentation and the subsequent production of protective short-chain fatty acids (SCFAs) [63].

While obesity directly heightens the risk of developing CKD, UPFs serve as key dietary contributors to this epidemic by promoting passive overconsumption and elevating caloric intake [13]. For instance, rats maintained on a cafeteria diet consisting of UPFs developed an obese phenotype accompanied by altered circulating fatty acid profiles, most notably an increase in total saturated fatty acids. This dietary model simultaneously drove gut microbial dysbiosis, suppressing beneficial Bacteroidetes populations [64].

A broad spectrum of ultra-processed food (UPF) components alters gut microbial composition and metabolic activity, directly fueling dysbiosis and mucosal inflammation. For example, maltodextrin—a food texturizer and sweetener—directly promotes cellular adhesion and biofilm formation across multiple *E. coli* strains, including adherent-invasive *E. coli*, mirroring the dense pathogenic biofilms observed in Crohn’s disease [65]. Similarly, dietary emulsifiers frequently added to processed goods represent major drivers of mucosal barrier degradation. In vitro investigations assessing common emulsifiers, such as propylene glycol monostearate (PGMS) and sodium stearoyl lactylate (SSL), revealed substantial shifts in the human fecal microbiota. Notably, SSL—commonly found in bakery products, coffee creamers and desserts—markedly depleted key butyrate-producing families (Clostridiaceae, Lachnospiraceae, and Ruminococcaceae) while promoting the expansion of Bacteroidaceae and Enterobacteriaceae [66]. Furthermore, an in vitro study by Han et al. demonstrated that the microbial breakdown of carrageenan, a food additive widely utilized for its gelling, thickening, and stabilizing properties, into degraded carrageenans significantly upregulated the production of key inflammatory mediators, including nitrite (NO_2_^−^), cyclooxygenase-2 (COX-2), and proinflammatory cytokines (IL-1, TNF-, and IL-6), thereby implicating a potential pathway in the pathogenesis of inflammatory bowel disease [67].

In CKD, a destructive cycle operates along the gut–kidney axis: proteolytic-derived microbial metabolites function as prominent circulating uremic toxins. Intestinal dysbiosis selectively promotes the proliferation of pathogenic bacteria harboring enzymes capable of generating key uremic toxins, such as indoxyl sulfate (IS), *p*-cresyl sulfate (pCS), indole-3-acetic acid (IAA), and trimethylamine *N*-oxide (TMAO). Due to impaired renal excretion, these metabolites accumulate systemically, exacerbating CKD pathology [30]. Increased levels of the Clostridiaceae and Peptostreptococcaceae families in the microbiota are directly associated with elevated human blood levels of TMAO. Because its excretion depends heavily on kidney function, plasma TMAO levels accumulate in CKD patients, directly correlating with a higher risk of all-cause mortality [68,69].

UPFs induce gut dysbiosis by depleting microbial heterogeneity and promoting opportunistic pathogens. This microbial imbalance disrupts intestinal epithelial integrity, enabling the translocation of bacterial endotoxins into the systemic circulation and triggering chronic inflammation via NF-κB activation [70]. Additionally, dysbiosis drives the structural degradation of epithelial tight junctions, provoking a “leaky gut” phenotype. This barrier compromise permits the paracellular translocation of bacterial lipopolysaccharides (LPSs) into the portal circulation, inducing systemic immune dysregulation and chronic low-grade inflammation [31].

### 4.6. Insulin Resistance and Metabolic Syndrome

The consumption of UPFs is a primary driver of global obesity, addictive eating behaviors and associated non-communicable diseases [71,72]. This dietary shift promotes cardiovascular–kidney–metabolic issues. Epidemiological evidence reveals a dose-dependent relationship between high UPF intake and metabolic syndrome (MetS). Cross-sectional analyses show that individuals in the highest quartile of UPF consumption face more than triple the odds of developing MetS compared to those in the lowest (OR = 3.27) [32]. Data from the ELSA-Brasil cohort support this, demonstrating an up to 1.3-fold higher incidence of MetS among the highest consumers, including a 19% increased risk (RR = 1.19) that remains significant even after adjusting for body mass index [73,74].

These dietary habits are disrupting intestinal barrier function and lipid metabolism, leading to low-grade inflammation, dyslipidemia and glucose intolerance [75]. Moreover, UPF-heavy diets promote hepatic lipogenesis and excess visceral adiposity, which culminates in systemic insulin resistance (IR). This systemic IR acts as an independent driver of CKD risk in non-diabetic patients, often manifesting early in mild-to-moderate CKD when GFR is still clinically preserved [75,76]. This creates risk for populations already vulnerable to microvascular complications—such as those with prediabetes and diabetes [33,77,78].

Obesity and visceral adiposity elevate the risk of proteinuria, progressive CKD and ESKD in both baseline-healthy individuals and high-risk patients [33,78]. UPF-driven systemic insulin resistance triggers compensatory hyperinsulinemia that upregulates the intrarenal renin–angiotensin–aldosterone system (RAAS) and enhances proximal tubular sodium reabsorption [78]. This leads to glomerular hyperfiltration. Ectopic lipid deposition and dysregulated adipokine signaling provoke chronic tubulointerstitial inflammation. Prospective findings from the Lifelines Cohort Study show that high UPF intake elevates the risk of incident CKD by 13% to 27% [33].

### 4.7. Soft Drinks: High-Fructose Corn Syrup and Phosphate Additives

Epidemiological data strongly links soft drinks to the pathogenesis of CKD due to two primary metabolic disruptors: high-fructose corn syrup (HFCS) and inorganic phosphate additives. The hepatic metabolism of fructose uniquely depletes intracellular adenosine triphosphate (ATP), which accelerates nucleotide degradation and upregulates uric acid synthesis [78]. This hyperuricemia drives renal injury through multiple pathways, including lipotoxicity and the direct promotion of tubulointerstitial inflammation [79]. Furthermore, fructose increases the urinary excretion of calcium and uric acid, significantly elevating the risk of nephrolithiasis, an independent risk factor for CKD [78].

Flavored beverages are based on inorganic phosphoric acid, used to enhance shelf life and taste. Unlike organic phosphorus, which is poorly absorbed, inorganic phosphate boasts an intestinal absorption rate of 90% to 100% [79]. This rapid absorption aggravates hyperphosphatemia, leading to vascular calcification, secondary hyperparathyroidism and finally renal function [79].

### 4.8. Artificial Sweeteners and ROS-Mediated Renal Damage

Artificial sweeteners such as aspartame act as metabolic disruptors that amplify systemic oxidative stress. In the intestinal tract, aspartame is metabolized into aspartic acid, phenylalanine and methanol [80]. This combination induces cellular oxidative stress by disrupting the balance between ROS production and the cell’s antioxidant compensatory systems [80]. This triggers lipid peroxidation, damaging the polyunsaturated fatty acids (PUFAs) critical to cellular membrane integrity [80].

Furthermore, artificial sweeteners have an impact on cellular lipid homeostasis, characterized by an accumulation of triacylglycerides and phospholipids, which operate synergistically with elevated ROS, leading to the systemic inflammation and progressive structural changes in renal tissue [80].

## 5. Epidemiological Evidence and Clinical Outcomes

### 5.1. Ultra-Processed Food Intake and Chronic Kidney Disease Incidence

A systematic review and meta-analysis by Leonberg et al. (2025) including seven cohort studies found that the higher UPF intake led to a 19% (HR, 1.19; 95% CI: 1.07–1.032) increased risk of developing CKD than in those with lower consumption [7]. The publication incorporated cohort studies from different countries (United States, Spain, the Netherlands, United Kingdom, China, and Korea), providing evidence from diverse populations. One of the largest prospective cohort studies included in this meta-analysis was conducted in the Netherlands, which contained over 70,000 participants without chronic kidney disease at the baseline. They were divided into quartiles according to the proportion of UPF intake in the diet (Q1 representing the lowest intake and Q4 representing the highest intake). At the beginning, dietary intake was evaluated with a validated food frequency questionnaire (FFQ). Subsequently, multivariable regression analysis was used to present the association between the proportion in grams/day of UPFs in the total diet and composite kidney outcomes, which were defined as incidence of CKD or a ≥30% estimated glomerular filtration rate decline. The results showed that participants in the highest quartile (Q4) had 27% increased odds of adverse kidney outcomes (OR, 1.27, CI %, 1.09–1.47) compared to the lower quartiles [81]. Similar findings were observed in another prospective cohort study conducted in the United States including over 14,000 participants without CKD at the baseline. The individuals in the highest quartile of UPF consumption had a 24% higher risk (HR, 1.24; 95% CI, 1.15–1.35) of developing CKD compared to the first quartile (Q1) [19]. Overall, the findings from all prospective cohorts in this meta-analysis suggest a consistent association between a higher UPF intake and an increased incidence of CKD. However, the results should be interpreted with caution due to substantial between-study heterogeneity (*I*^2^ = 80.4%, *p* < 0.001), differences in dietary assessment methods, and the limited ability to account for changes in dietary patterns over time [7,19,81].

The association between UPF intake and incident CKD was also taken into consideration in another recent meta-analysis conducted by Kermani et al., which included 33 observational studies (cross-sectional, cohort, or case–control studies) between 2007 and 2024 across multiple nations. A total of 786,216 participants were included, and the pooled analysis demonstrated that greater UPF consumption led to a 16% higher risk of developing CKD (RR, 1.16; 95% CI: 1.09–1.23). Subgroup analysis confirmed that this relationship remained prominent across different geographical regions; however, studies conducted in USA and Asian countries had a more pronounced link than those in Europe [82]. Among individual cohort studies contained in this meta-analysis, several of them reported significant evidence. For instance, Gu et al. investigated the association between UPF intake and CKD development based on two large prospective cohorts: TCLSIH and UK Biobank. In total, 23,775 and 102,332 participants without any kidney dysfunctions were included in this research. At the beginning, the consumption of UPF was assessed by a validated food frequency questionnaire in TCLSIH and 24 h dietary recalls in the US Biobank cohort; then, the individuals were divided into quartiles according to the increasing consumption of UPF (Q1–Q4). In both cohorts, a higher UPF intake had a greater risk of CKD across quartiles Q1–Q4 based on multivariable HRs (95% CI). Importantly, in the TCLSIH cohort, the highest quartile had a 58% greater likelihood of CKD (HR, 1.58; 95% CI: 1.07–2.34), while the highest quartile in US Biobank cohort was associated with a 25% higher risk (HR, 1.25; 95% CI: 1.09–1.43) [83].

Furthermore, it is also noteworthy that this meta-analysis revealed a dose–response correlation, demonstrating that an increase in UPF intake of about 1 portion/day was associated with a 5% increased risk of CKD (RR, 1.05; 95% CI: 1.02–1.09). Despite the inherent limitations of this meta-analysis, including variation in age, sex, body mass index (BMI), energy intake, educational level, smoking status, and others, the overall findings support a higher UPF intake as a potential risk factor for CKD development [82,83].

Table 3 summarizes the main findings of the two meta-analyses described in this section.

### 5.2. Association Between Ultra-Processed Food and Kidney Markers

While the eGFR reflects the actual filtration capacity of the kidney responding to the loss functional nephrons, albuminuria indicates podocyte and glomerular barrier damage. These criteria are also used to determine staging for CKD in epidemiological studies, which rely on monitoring the dynamics of these parameters [84,85]. Both markers are pivotal for assessing the risk of developing CKD [86].

In the aforementioned Lifelines study from the Netherlands, it was also demonstrated that a higher consumption of UPF accelerated the decline in eGFR. The participants from the highest quartile of UPF consumption had a more rapid eGFR decline. This inverse association was further supported by regression analysis (β, −0.17; 95% CI: −0.23 to −0.17), which indicated the impact of UPF intake on decreasing renal function. Moreover, the authors also estimated dose–response association—every 10% increment in UPF consumed in the diet was related to an 11% higher risk of the composite outcome (OR, 1.11; 95% CI: 1.06–1.17) and faster eGFR decline (0.06 mL/min/1.73 m^2^ per year) [81].

Another prospective cohort study conducted by Rey Garcia et al. demonstrated the relationship between UPF intake and kidney function, which was measured based on fluctuations in serum creatinine (SCr) and eGFR. A total of 1312 participants were distributed to the tertiles according to the growing total energy intake from UPFs. The total adjusted odds ratio of kidney dysfunction across tertiles was 1.56 for the second tertile and 1.74 for the highest tertile, which emphasizes the necessity of considering UPFs as a risk factor in the context of renal dysfunction development [87].

Vale-Hita et al. demonstrated the possible correlation between UPF intake and kidney dysfunction in the older adult population with overweight/obesity and metabolic syndrome based on cystatin C. A total of 1851 participants were incorporated in the cross-sectional analysis and 1700 participants in the longitudinal analysis. The mean age of these individuals was 65 ± 5 years. UPF consumption showed a statistically inverse association with eGFR (β, −2.72 mL/min/1.73 m^2^; 95% CI: −4.89 to −0.54). The research revealed that every 10% increment in UPF consumption was associated with a decline in the eGFR (β, −1.35 mL/min/1.73 m^2^; 95% CI: −2.74 to −1.00). The results were compatible in longitudinal studies. Participants in the highest tertile of UPF consumption after one-year of follow-up had a greater decline in eGFR (β = −1.45 mL/min/1.73 m^2^) as well as after 3-years follow-up (β, −2.18 mL/min/1.73 m^2^) [88].

Overall, the presented findings show that consumption of UPFs and renal function decline may be correlated with each other. However, the magnitude and consistency of these associations may vary across populations, particularly according to age, baseline kidney function, the presence of comorbidities, and methods used to assess dietary intake [87,88].

### 5.3. The Impact of Ultra-Processed Food Consumption on Progression of Chronic Kidney Disease

According to KDIGO, CKD is classified based on eGFR (G1–G5). Within this methodological framework, stage G5 of KDIGO classification, called ESKD, corresponds to eGFR < 15 mL/min/1.73 m^2^, or a situation where a patient requires renal replacement therapy. In addition, it also includes staging based on the level of albuminuria (A1, A2, A3) by estimating ACR in early morning samples of urine [89]. The progression of kidney damage is predicted by a multitude of risk factors, which can be categorized as non-modifiable or modifiable. There are several categories, including genetic factors; geographic factors; individual factors such as age, sex, birth weight, familiarity of kidney disease, and pregnancy; cardiovascular factors; and lifestyle factors such as physical activity, smoking, socioeconomic status, and diet (high sodium intake, unhealthy fats, and processed foods). The latter may contribute to hypertension and metabolic abnormalities, and also increase the risk of CKD progression [90].

Among the available studies investigating the impact of UPF consumption on CKD progression, the prospective cohort study conducted by Sullivan et al. presented significant evidence. It was a multicenter study (*n* = 3939) consisting of adults (21–74 years) with reduced eGFR at the baseline (eGFR 20–70 mL/min/1.73 m^2^). The dietary intake was estimated by a validated food frequency questionnaire—the Diet History Questionnaire (DHQ). Follow-up assessments were conducted every 6 months. There were 1047 reported events of CKD progression during 7 years of follow-up, which was defined as a ≥50% decline in the estimated glomerular filtration rate or initiation of kidney replacement therapy. A higher UPF consumption was associated with a 33% greater risk of CKD progression compared to tertile 3 to tertile 1. After including factors such as demographic characteristics, lifestyle, energy intake, and study site, the results were similar comparing tertile 3 to tertile 1 (HR, 1.22; 95% CI: 1.04–1.42). What is more, among individuals with CKD stage 3a to 5, there was no association between UPF intake and CKD progression (tertile 3 vs. tertile 1; HR, 1.12; 95% CI: 0.95–1.32); however, participants at stages 1–2 had a higher risk (tertile 3 vs. tertile 1; HR, 2.61; 95% CI: 1.32–5.18) [91].

Another valuable prospective cohort study was presented by Kurniawan et al., for which 41,128 adults (40–95 years) with eGFR < 90 mL/min/1.73 m^2^ and albuminuria at the baseline were recruited. Dietary assessment was evaluated by using a semi-quantitative food frequency questionnaire (SQ-FFQ). Patients with increased consumption of processed meat and lower intake of plant-based food had a higher risk of CKD progression (HR, 1.15; 95% CI: 1.02–1.29) [20].

The available evidence suggests that increased ultra-processed food consumption may be associated not only with development of CKD but also with faster progression of kidney dysfunction [20,91]. However, the observed association might depend on baseline kidney function, as the correlation was more pronounced in patients with earlier stages of CKD and was not significantly observed in those at more advanced stages. Therefore, these findings should be interpreted cautiously and do not support that reducing UPF intake directly prevents CKD progression [91].

### 5.4. Ultra-Processed Food and the Risk of Cardiovascular Complications and All-Cause Mortality in Populations with Chronic Kidney Disease

CKD is not just recognized as an isolated renal pathology, but also as an independent predictor of severe outcomes such as CVD and all-cause mortality. Epidemiological studies have demonstrated that a worsening kidney function estimated by a reduced eGFR and increasing albuminuria is closely associated with biochemical disturbances, hypertension, severe cardiovascular disease, insulin resistance, and even risk of death [85,92]. The leading cause of death for patients with stages 4–5 CKD is CVD rather than ESKD. It is related to the proinflammatory state contributing to vascular and myocardial remodeling processes, which lead to atherosclerosis lesions, vascular and myocardial calcification, and then fibrosis. As a result, patients often succumb to cardiovascular complications prior to the onset of CKD stages involving dialysis initiation [93]. In the Framingham Offspring Study, the highest quartile of UPF consumption had a greater risk of CVD incidence (per 1000 person years; 3.36 vs. 6.64) [94]. Therefore, modifiable factors such as dietary habits should be always evaluated to reduce these problems [93,94].

As mentioned in the previous section, Sullivan et al. extended their research beyond CKD progression by evaluating the association between UPF intake and the risk of cardiovascular incidents and all-cause mortality in patients with renal kidney dysfunction. Over 12 years of follow-up, 406 participants developed CVD. Individuals in the highest tertile of UPF intake had a 21% higher risk of all-cause mortality compared with those in the lowest tertile. Moreover, each additional UPF portion was associated with a 7% higher risk of mortality (HR, 1.07; 95% CI: 1.04–1.10) [91]. In addition, Kurniawan et al. also demonstrated that a kidney function-related dietary pattern characterized by a high intake of processed foods was associated with 10–31% increased odds of cardiovascular incidence and a greater risk of moderate/severe kidney dysfunction (OR, 1.15; 95% CI: 1.02–1.29) [20].

Notably, the Moli-Sani study suggested that impaired renal function may constitute the potential link between UPF intake and CVD. In patients with cardiovascular disease, a higher UPF consumption was associated with increased mortality risk, and also with worsening of renal markers such as cystatin C, which may indicate kidney dysfunction. A higher level of cystatin C could potentially explain 18.8% and 16.6% of the association between UPF and all-cause mortality and cardiovascular incidences [95].

Taken together, the current evidence supports a possible association between higher UPF consumption and unfavorable renal and cardiovascular outcomes [20,91,95]. These findings provide a rationale for evaluating the effectiveness of reducing UPF intake in dietary patterns, especially in patients with potential kidney and cardiovascular dysfunction [96].

## 6. Dietary Approaches in Chronic Kidney Disease: Guidelines, Dietary Patterns, and Clinical Relevance

### 6.1. Main Findings and Clinical Relevance

CKD is a multifactorial condition whose progression is strongly influenced by modifiable lifestyle factors, including dietary habits. The available evidence indicates that a high intake of UPFs is consistently associated with an increased risk of CKD incidence, accelerated decline in kidney function, higher prevalence of albuminuria, and greater risk of ESKD and mortality [36,97,98]. Although the majority of available studies are observational and cannot establish causality, the consistency of associations across different populations supports the hypothesis that higher UPF consumption may represent a modifiable dietary risk factor for adverse kidney outcomes [36,99].

However, these associations should be interpreted cautiously because of residual confounding, possible reverse causality, and differences in dietary assessment methods [12,96]. Individuals with higher UPF consumption may also differ in socioeconomic status, lifestyle, comorbidities, and overall dietary quality. Therefore, the available evidence does not establish that UPFs independently or directly cause CKD progression [12,96].

The potential clinical relevance of UPFs should be considered in the context of overall dietary quality rather than individual nutrients alone. UPFs commonly contribute to higher intakes of sodium, added sugars and energy-dense foods, while displacing minimally processed foods. Thus, reducing UPF consumption may be a useful component of dietary management in CKD, but it should not be considered an isolated intervention [12,13].

An additional limitation concerns the NOVA classification, which may be affected by heterogeneity within food categories and potential misclassification of individual products [100]. These limitations may contribute to exposure misclassification and should be considered when interpreting epidemiological associations [100,101].

From a clinical perspective, dietary assessment in CKD may therefore benefit from considering both overall dietary quality and the degree of food processing. Nevertheless, randomized controlled trials evaluating whether reducing UPF intake improves kidney outcomes remain scarce. Future studies should determine whether targeted UPF reduction improves eGFR, albuminuria, and CKD progression and whether the observed associations are causal [102].

### 6.2. Dietary Guidelines

Current nutritional recommendations for CKD emphasize individualized medical nutrition therapy (MNT) based on CKD stage, kidney function, nutritional status, treatment modality, metabolic abnormalities, comorbidities, and cardiovascular risk [2,103]. The primary goals of dietary management are to maintain adequate nutritional status, prevent protein–energy wasting, improve metabolic control, reduce cardiovascular risk, and potentially slow CKD progression as part of comprehensive multidisciplinary care [2,103].

The 2024 KDIGO guideline, supported by the 2020 Kidney Disease Outcomes Quality Initiative (KDOQI) Clinical Practice Guideline for Nutrition in CKD, recommends limiting sodium intake to <2 g/day (equivalent to <5 g/day of sodium chloride) in adults with CKD [2,103]. Sodium restriction improves blood pressure control and may reduce albuminuria, particularly when combined with appropriate pharmacological therapy, thereby contributing to comprehensive CKD management [2,103]. These recommendations are consistent with the WHO guidance on sodium reduction for the general population [104,105].

Dietary protein intake should be tailored according to CKD stage, metabolic stability, nutritional status, and kidney replacement therapy. In metabolically stable adults with CKD stages G3–G5 who are not receiving dialysis, KDIGO recommends a protein intake of approximately 0.8 g/kg body weight/day while avoiding a high protein intake (>1.3 g/kg body weight/day), particularly in individuals with increased risk of CKD progression [2,103]. Protein restriction should be accompanied by adequate energy intake and regular nutritional assessment to minimize the risk of protein–energy wasting. In contrast, patients receiving maintenance dialysis generally require a higher protein intake because of increased protein catabolism and amino acid losses associated with dialysis treatment [2,103]. Preserving nutritional status remains a key therapeutic objective across all CKD stages, as protein–energy wasting is consistently associated with increased morbidity and mortality [103,106].

Current cardiovascular prevention guidelines from the European Society of Cardiology (ESC) and the American Heart Association (AHA) recommend dietary patterns rich in vegetables, fruits, whole grains, legumes, nuts, and unsaturated fats while limiting foods high in sodium, added sugars, and saturated fats [107,108]. In patients with CKD, these dietary patterns should be adapted according to disease stage, biochemical parameters, and individual clinical characteristics [2,103]. Contemporary guidelines increasingly advocate for a whole-diet approach that emphasizes overall dietary quality rather than isolated nutrient restriction [2,103,108]. Mediterranean-style and predominantly plant-based dietary patterns may provide cardiovascular and metabolic benefits and have been associated with improved kidney outcomes in observational studies, although high-quality randomized evidence remains limited [2,103,109,110]. Rather than routinely restricting potassium or phosphorus intake, current recommendations support individualized assessment based on laboratory findings, dietary sources, medication use, and clinical status. Particular attention should be paid to reducing highly bioavailable phosphorus derived from food additives while avoiding unnecessary restriction of nutrient-rich foods [2,103].

Taken together, current evidence supports individualized MNT as an integral component of CKD management [2,103]. Whenever available, dietary care should be delivered by healthcare professionals with expertise in renal nutrition [2,103]. Personalized dietary interventions may improve blood pressure control, metabolic parameters, nutritional status, and cardiovascular risk while minimizing unnecessary dietary restrictions and supporting long-term kidney health [2,103,110].

### 6.3. Dietary Patterns Relevant to Chronic Kidney Disease

Dietary interventions represent an important component of CKD management, with growing evidence suggesting that overall dietary patterns may be more relevant than isolated nutrient modifications. A whole-diet approach considers the synergistic effects of different nutrients and food components, providing a more practical strategy for nutritional counseling and disease management [103,111]. Healthy dietary patterns characterized by a high consumption of fruits, vegetables, whole grains, legumes, nuts, and fish, together with reduced intake of red and processed meats, have been associated with a lower risk of CKD development and more favorable kidney outcomes [18]. Several large cohort studies have demonstrated that greater adherence to high-quality dietary patterns, including the Mediterranean diet, The Dietary Approaches to Stop Hypertension (DASH) diet, and other healthy dietary indices, is associated with reduced risk of incident CKD, slower CKD progression, and lower all-cause mortality [112,113]. The potential benefits of these dietary patterns may be related to their effects on multiple mechanisms involved in CKD pathogenesis, including inflammation, oxidative stress, metabolic disturbances, and dietary acid load [114]. Consequently, Mediterranean, DASH, and plant-based dietary patterns have gained increasing attention as potential strategies to support kidney health [103,114]. However, nutritional interventions in patients with established CKD require individualization due to possible restrictions related to protein, potassium, and phosphorus intake [114].

#### 6.3.1. The Mediterranean Diet

The Mediterranean diet is a whole-diet strategy characterized by a high intake of minimally processed foods, including vegetables, fruits, legumes, whole grains, fish, and extra virgin olive oil (EVOO), while limiting red meat and ultra-processed products. Its potential benefits may result from the combined effects of these dietary components, including anti-inflammatory, antioxidant, antihypertensive, and cardioprotective properties, influencing not only kidney outcomes, but also common cardiometabolic complications [115,116].

A high fiber intake may also promote a favorable gut microbiota composition, increase short-chain fatty acid production, and reduce the generation of gut-derived uremic toxins such as indoxyl sulfate, *p*-cresyl sulfate, and TMAO [117]. Additionally, Noce et al. demonstrated that daily consumption of EVOO, which is rich in minor polar compounds, improved renal function biomarkers, inflammatory status, oxidative stress, and lipid profiles in CKD patients, supporting the potential role of EVOO as one of the components contributing to the effects of the Mediterranean diet [116].

Clinical evidence, however, is not entirely consistent and is largely derived from observational studies. Huang et al. found that higher adherence to the Mediterranean diet was associated with a lower risk of all-cause mortality in 1110 elderly men, although no significant association with the rate of kidney function decline was observed, suggesting that the survival benefit may be largely related to improvements in cardiovascular risk factors rather than a direct effect on preservation of kidney function [118]. In kidney transplant recipients, higher Mediterranean diet adherence was independently associated with a lower risk of graft failure, kidney function decline, and graft loss, suggesting potential benefits for long-term renal outcomes after transplantation [119]. Similarly, Chrysohoou et al. reported lower serum urea and creatinine levels and increased creatinine clearance among individuals with greater Mediterranean diet adherence [120]. Furthermore, a recent meta-analysis suggested that this diet may slow kidney function decline in patients with non-dialysis CKD, particularly those with mild-to-moderate disease, and also reported lower blood urea nitrogen, improved body composition, reduced CRP levels associated with high-phenolic EVOO, and stable potassium and phosphorus concentrations [121].

Despite its potential benefits, implementation of the Mediterranean diet in CKD requires individualization according to disease stage, nutritional status, and biochemical parameters. The Mediterranean Renal (MedRen) diet has been proposed as an adaptation of the traditional Mediterranean pattern, which maintains its main characteristics while adjusting protein, sodium, and phosphate intake according to nephrology recommendations [122]. Therefore, the Mediterranean diet may represent a flexible dietary approach in CKD, provided that it is appropriately adapted to the specific nutritional requirements of individual patients [103,122].

#### 6.3.2. The DASH Diet

The DASH diet is characterized by a high intake of fruits, vegetables, whole grains, legumes, nuts, and low-fat dairy products, together with reduced consumption of sodium, saturated fats, and total fat [123,124]. Initially developed for blood pressure control, the DASH diet has demonstrated beneficial effects on vascular function. Its potential relevance to CKD lies in its favorable effects on blood pressure and cardiovascular risk, as well as its high content of plant-derived foods and fiber and lower sodium and dietary acid load [123,125,126]. These characteristics may also influence metabolic, inflammatory, oxidative, and gut-microbiota-related pathways implicated in CKD progression [126]. However, implementation of the DASH diet in patients with established CKD requires individualization, particularly regarding potassium, phosphorus, and protein intake, as nutritional requirements may vary depending on disease stage and nutritional status [124,126].

Several observational studies have investigated the relationship between DASH adherence and CKD outcomes. Mozaffari et al., in a systematic review and meta-analysis including seven observational studies and more than 568,000 participants, reported that higher adherence to the DASH diet was associated with a lower risk of CKD development, with the protective association mainly observed in prospective cohort studies [127]. Similarly, Lee et al. analyzed 2408 elderly Korean adults and found that higher DASH adherence was associated with lower odds of CKD, while a higher fiber intake was independently associated with reduced CKD prevalence [128]. Among patients with established CKD, Banerjee et al. found that lower DASH adherence was associated with a higher risk of ESRD in adults with moderate CKD and hypertension [129]. However, a meta-analysis of prospective CKD cohorts found that although higher DASH adherence was associated with a greater improvement in eGFR and lower urinary ACR, the evidence was insufficient to demonstrate a statistically significant effect on CKD progression [130].

Beyond renal outcomes, the cardiovascular effects of the DASH diet may represent an important component of its potential role in CKD management. Tyson et al. demonstrated that higher DASH adherence was associated with lower systolic and diastolic blood pressure among Black American adults with CKD, supporting its potential cardiovascular relevance [125]. Additionally, findings from the CRIC study showed that adherence to DASH and other healthy dietary patterns was associated with a lower risk of CKD progression and all-cause mortality, supporting the importance of overall dietary quality rather than isolated nutrient restriction [99].

Overall, the DASH diet represents a potentially beneficial dietary approach in CKD, combining favorable effects on blood pressure control, cardiometabolic risk factors, and potentially kidney outcomes. Nevertheless, similarly to other dietary interventions for CKD, its application requires adaptation to individual patient characteristics and disease stage to balance potential benefits with nutritional safety considerations [124,131].

#### 6.3.3. Plant-Based Diet

Plant-based dietary patterns have gained increasing interest in CKD management due to their potential metabolic and cardiovascular benefits. They are characterized by a high intake of minimally processed plant foods, including whole grains, fruits, vegetables, legumes, nuts, and seeds, which provide greater amounts of dietary fiber, antioxidants, unsaturated fatty acids, and alkali-producing nutrients, while reducing the intake of saturated fats and highly bioavailable phosphorus [132,133]. Importantly, the term “plant-based diet” encompasses a broad spectrum of dietary patterns, and the potential benefits appear to depend primarily on the quality of consumed plant foods rather than their plant origin alone [134,135].

The potential renoprotective effects of healthy plant-based diets may involve several mechanisms relevant to CKD progression. Higher fiber intake and greater consumption of plant-derived foods may contribute to favorable changes in gut microbiota composition, reduced production of gut-derived uremic toxins, decreased DAL, and attenuation of inflammation and oxidative stress [132,133]. Additionally, plant-based dietary patterns may support improved blood pressure control, phosphorus homeostasis, and metabolic acidosis management, which may contribute to slower CKD progression [132,136].

Evidence from observational studies suggests that the quality of plant-based dietary patterns is a key determinant of their effects on kidney outcomes. Dang et al. demonstrated in a systematic review and meta-analysis including over 120,000 participants that higher adherence to plant-based diets was associated with a lower risk of CKD development and slower disease progression, with the benefits primarily observed for healthy plant-based patterns [137]. Similarly, analyses from the CRIC study showed that adherence to a healthy plant-based diet was associated with improved survival, whereas unhealthy plant-based dietary patterns, rich in refined grains, sugar-sweetened beverages, sweets, and other processed plant foods, were associated with worse renal outcomes and higher mortality risk [134]. Findings from the UK Biobank cohort further supported this distinction, demonstrating that higher adherence to a healthful plant-based diet was associated with lower all-cause mortality among individuals with CKD, while lower-quality plant-based patterns did not provide the same benefits [135]. Thus, replacing animal foods with refined or ultra-processed plant products should not necessarily be assumed to confer the same benefits as increasing minimally processed plant foods.

An important aspect of plant-based dietary strategies in CKD is the concern regarding potassium restriction. However, several reviews suggest that routine limitation of fruits and vegetables may not be necessary, and potassium intake should be individualized according to CKD stage and serum potassium levels rather than broadly restricting plant foods [132,138]. With appropriate monitoring, the potential benefits of plant-based diets may outweigh the risks associated with hyperkalemia in selected patients [133,138].

A specific adaptation of plant-based nutrition for CKD is the plant-dominant low-protein diet (PLADO), which combines moderate protein restriction (0.6–0.8 g/kg/day) with more than 50% of protein derived from plant sources, alongside an emphasis on minimally processed foods, adequate energy intake, high fiber consumption, and sodium restriction [139]. By combining greater consumption of plant foods with controlled protein intake, PLADO has been proposed as a patient-centered strategy for conservative CKD management, although further clinical evidence is needed to establish its long-term efficacy [139,140].

#### 6.3.4. Low-Protein Diet

Protein restriction remains an important but debated component of nutritional management in non-dialysis CKD. A low-protein diet (LPD) is commonly defined as a dietary protein intake of approximately 0.6 g/kg/day, representing a reduction from the recommended intake of 0.8 g/kg/day [141,142]. Its potential renoprotective effects have been attributed to reduced intraglomerular pressure and hyperfiltration, as well as lower nitrogenous waste accumulation, dietary acid load, uremic toxin generation, and metabolic burden [141,143,144,145].

Evidence regarding the effectiveness of LPDs in slowing CKD progression remains complex. Although randomized controlled trials have not consistently demonstrated a significant benefit of protein restriction in unselected CKD populations, observational data suggest that appropriately tailored dietary interventions may provide renal benefits in selected patients [146]. Brum et al., in a cohort of 438 patients with CKD, reported that adherence to an LPD (<0.8 g/kg/day) was associated with a slower decline in eGFR compared with non-adherence, suggesting that sustained adherence to protein targets may contribute to preservation of kidney function over time [147]. Similarly, reviews by Ko et al. and Kim and Jung indicate that protein restriction may delay CKD progression, although its effectiveness depends on appropriate patient selection and careful nutritional monitoring [143,144]. These findings support the possibility that sustained adherence, rather than protein restriction alone, may be a key determinant of clinical benefit.

A major challenge associated with protein restriction is the potential risk of protein–energy wasting and loss of muscle mass, particularly among older adults and patients with impaired nutritional status. Piccoli et al. emphasize that indiscriminate protein restriction may be harmful in individuals at risk of malnutrition, and that dietary protein restriction should mainly be considered in patients with progressive CKD and preserved nutritional status [148]. Therefore, current approaches have shifted from universal protein restriction toward personalized dietary strategies that consider CKD stage, nutritional status, comorbidities, patient preferences, and the risk of malnutrition [141,146,148].

More intensive protein restriction can be achieved with very-low-protein diets (VLPDs), typically providing approximately 0.3–0.4 g/kg/day of protein and often supplemented with ketoanalogues of essential amino acids to maintain adequate amino acid availability while reducing nitrogen load [145,149]. VLPDs may provide additional benefits by reducing nitrogenous waste accumulation, dietary acid load, phosphate burden, and gut-derived uremic toxin production [145].

In patients with advanced CKD, Bellizzi et al. reported improved blood pressure control following an anketoanalogue-supplemented VLPD, although the effect could not be attributed to protein restriction alone, given concomitant reductions in sodium intake and increased use of plant-based proteins [149]. Thus, the potential benefits of VLPDs may result from a combination of reduced protein intake and wider modifications of dietary composition.

Overall, the available evidence supports protein restriction as a potentially useful but highly individualized component of CKD management. Rather than focusing solely on reducing protein quantity, current approaches emphasize optimizing the amount and source of protein while preserving nutritional status [141,146].

Taken together, dietary quality represents an important component of CKD prevention and management, with healthy Mediterranean, DASH, and plant-based dietary patterns associated with more favorable kidney outcomes [150,151]. Importantly, the potential benefits appear to depend not only on the dietary pattern itself but also on the quality of consumed foods, appropriate patient selection, and individual nutritional requirements [151,152]. Dietary management should therefore focus on improving overall diet quality while balancing potential renal and cardiovascular benefits against risks such as malnutrition and electrocyte disturbances. Importantly, the effectiveness of dietary recommendations in clinical practice may also be limited by their accessibility and affordability, as minimally processed foods may be less available or more costly for some patients. Future approaches should therefore combine personalized nutritional strategies with consideration of patients’ nutritional status, preferences, and real-world socioeconomic barriers. Dietary recommendations and dietary patterns relevant to CKD management are summarized in Table 4. 

## 7. Conclusions

UPFs are typically high in sodium, added sugars, saturated fats, and highly bioavailable phosphate and potassium additives, while providing relatively little dietary fiber, vitamins, and other protective nutrients. Current evidence indicates that individuals with the highest UPF intake have an approximately 16–19% greater risk of incident CKD than those with the lowest intake. Higher consumption has also been associated with faster eGFR decline, progression of existing kidney dysfunction, and increased cardiovascular and all-cause mortality.

The adverse renal effects of UPFs are likely mediated through several interconnected mechanisms, including inflammation, oxidative stress, sodium overload, metabolic acidosis, gut microbiota dysbiosis, insulin resistance, hypertension, and obesity. These associations have been observed in both generally healthy populations and individuals with metabolic disorders or other comorbidities. The relationship with CKD progression may be particularly relevant in earlier disease stages, although findings in advanced CKD remain less consistent.

Reducing UPF intake and replacing these products with minimally processed foods, especially vegetables, fruits, legumes, whole grains, and other plant-based foods, may support kidney protection, improve blood pressure and metabolic control, and reduce cardiovascular risk. Mediterranean, DASH, and healthy plant-based diets appear particularly beneficial. However, dietary management should be individualized according to CKD stage, kidney function, nutritional status, laboratory findings, comorbidities, and treatment modality. Sodium intake should generally remain below 2 g/day, while a protein intake of approximately 0.8 g/kg/day is recommended for metabolically stable patients with non-dialysis CKD stages G3–G5. More restrictive diets require careful monitoring to prevent protein–energy wasting.

Overall, diet quality appears to be more important than restriction of individual nutrients alone. Nutritional care should preferably involve professionals experienced in renal nutrition and should avoid unnecessary potassium or phosphorus restrictions. Nevertheless, most available evidence is observational, and differences in UPF definitions, dietary assessment methods, study populations, and renal outcomes limit causal interpretation. Further prospective studies and randomized clinical trials are needed to determine whether reducing UPF consumption directly slows CKD progression and to establish stage-specific dietary recommendations.

## Figures and Tables

**Table 1:** NOVA food classification system.

NOVA Group	Food Category	Brief Description	Examples	Potential Relevance to CKD	References
1	Unprocessed or minimally processed foods	Natural foods altered only by basic processes such as cleaning, freezing, drying, and pasteurization.	Fresh or frozen vegetables and fruits, grains, legumes, milk, eggs, meat, fish.	Better diet quality, higher fiber intake. May support kidney and cardiovascular health.	[2,15,17]
2	Processed culinary ingredients	Substances obtained from group 1 foods or natural sources and mainly used for meal preparation.	Oils, fats, sugar, salt.	Excessive intake of salt and fats may promote hypertension and metabolic risk, indirectly increasing CKD risk.	[15,18]
3	Processed foods	Foods produced by combining group 1 foods with group 2 ingredients, mainly using preservation methods such as canning or fermentation.	Canned vegetables, cheese, freshly prepared bread, salted nuts.	Some products may contain high amounts of sodium and phosphorus, contributing to hypertension, mineral disturbances, and potentially contributing to CKD progression.	[14,15]
4	Ultra-processed foods	Industrial formulation containing refined food-derived substances, additives, and little or no intact whole food.	Soft drinks, packaged snacks, processed meats, instant meals, sweetened breakfast cereal.	High amounts of sodium, added sugars, saturated fats, phosphate and potassium-containing additives, while low in fiber. Faster eGFR decline, progressive kidney dysfunction.	[13,14,15,19,20]

Abbreviations: UPFs, ultra-processed foods; CKD, chronic kidney disease.

**Table 2:** Pathophysiological mechanisms linking UPF consumption to CKD progression.

Mechanism	UPF-Related Factors	Pathophysiological Pathway	Renal Effect	References
Inflammation and oxidative stress	Refined carbohydrates, saturated fats, food additives, inorganic phosphates, low fiber and antioxidant content.	Increased intestinal permeability, ROS accumulation, and elevated inflammatory mediators, including CRP, IL-6 and TNF- α.	Endothelial dysfunction, glomerular hyperfiltration, tubulointerstitial fibrosis, albuminuria, decline in eGFR	[13,25]
Metabolic acidosis and dietary acid load	High intake of phosphorus, animal protein and acidogenic processed foods; low intake of base-producing plant foods.	Acid retention, increased ammoniagenesis, activation of endothelin-1, angiotensin II and aldosterone.	Tubulointerstitial inflammation, progressive nephron destruction, accelerated GFR decline	[13,26]
Hypertension and sodium overload	High sodium content and sodium-containing additives.	Impaired sodium excretion, fluid volume dysregulation, increased blood pressure, oxidative stress, proteinuria.	Progressive decline in kidney function and cardiovascular risk.	[27,28,29]
Gut microbiota dysbiosis	Low fiber intake, high saturated fat content and food additives.	Reduced beneficial bacterial population, increased production of IS, pCS, IAA and TMAO, impaired intestinal barrier and LPS translocation.	Accumulation of uremic toxins, systemic inflammation and worsening CKD pathology.	[30,31]
Insulin resistance and metabolic syndrome	High energy density, refined carbohydrates, added sugars and saturated fats.	Insulin resistance, hyperinsulinemia, RAAS activation, increased sodium reabsorption and dysregulated adipokine signaling.	Glomerular hyperfiltration, proteinuria, tubulointestinal inflammation, increased risk of CKD progression	[32,33]

Abbreviations: CKD, chronic kidney disease; CRP, *C*-reactive protein; eGFR, estimated glomerular filtration rate; IAA, indole-3-acetic acid; IS, indoxyl sulfate; LPSs, lipopolysaccharides; pCS, *p*-cresyl sulfate; RAAS, renin–angiotensin–aldosterone system; ROS, reactive oxygen species; TMAO, trimethylamine *N*-oxide; TNF-α, tumor necrosis factor-alpha; UPFs, ultra-processed foods.

**Table 3:** Main findings of the two meta-analyses on UPF consumption and the risk of developing CKD.

Meta-Analysis	No. of Studies	No. of Participants	Overall Association
Leonberg et al., (2025) [7]	7 prospective cohorts	511,589	HR, 1.19 (95% CI: 1.07–1.32)
Kermani et al., (2025) [82]	33 (cross-sectional, cohort, and case–control studies)	786,216	RR, 1.16 (95% CI: 1.09–1.23)

**Table 4:** Dietary recommendations and patterns relevant to CKD management.

Diet	Main Characteristics	Potential Relevance to CKD	Clinical Considerations	References
Mediterranean	Plant foods, fish, olive oil, limited red meat and UPFs.	May improve cardiovascular health and kidney outcomes; lower inflammation and mortality.	Adapt protein, sodium, potassium and phosphorus intake.	[115,118,121,122]
DASH	Fruits, vegetables, whole grains, low sodium intake.	Supports blood pressure control and may reduce CKD risk; potentially improved eGFR and albuminuria.	Individualize potassium, phosphorus and protein intake.	[124,127,130]
Healthy plant based	Minimally processed plant foods rich in fiber.	May reduce acid load, uremic toxins and CKD risk.	Benefits depend on food quality.	[132,134,137]
LPD	Approximately 0.6 g protein/kg/day.	Lower nitrogenous waste and intraglomerular pressure; possible slower eGFR decline	Risk of protein–energy wasting and muscle loss.	[141,147,148]
VLPD	Approximately 0.3–0.4 g/kg/day with ketoanalogues.	Lower nitrogen, acid, and phosphate burden; possible delayed dialysis.	Only for selected patients under specialist supervision.	[145,149]

Abbreviations: CKD, chronic kidney disease; DASH, Dietary Approaches to Stop Hypertension; eGFR, estimated glomerular filtration rate; LPD, low-protein diet; VLPD, very-low-protein diet.

## Data Availability

No new data was created or analyzed in this study. Data sharing is not applicable to this article.

## Abbreviations

The following abbreviations are used in this manuscript:

ACR	Albumin-to-creatinine ratio
AGEs	Advanced glycation end-products
AHA	American Heart Association
Ang II	Angiotensin II
ARIC	Atherosclerosis Risk in Communities
BMI	Body mass index
BP	Blood pressure
CKD	Chronic kidney disease
CRP	*C*-reactive protein
CVD	Cardiovascular disease
DAL	Dietary acid load
DASH	Dietary Approaches to Stop Hypertension
DHQ	Diet History Questionnaire
DII	Dietary Inflammatory Index
eGFR	Estimated glomerular filtration rate
ESC	European Society of Cardiology
ESKD	End-stage kidney disease
EVOO	Extra virgin olive oil
ET-1	Endothelin-1
FFQ	Food frequency questionnaire
IAA	Indole-3-acetic acid
IL	Interleukin
IR	Insulin resistance
IS	Indoxyl sulfate
KDIGO	Kidney Disease: Improving Global Outcomes
KDOQI	Kidney Disease Outcomes Quality Initiative
LPDs	Low-protein diets
LPS	Lipopolysaccharide
MedRen diet	Mediterranean Renal diet
MetS	Metabolic syndrome
MNT	Medical nutrition therapy
pCS	p-cresyl sulfate
PRAL	Potential renal acid load
RAAS	Renin–angiotensin–aldosterone system
ROS	Reactive oxygen species
SCr	Serum creatinine
SQ-FFQ	Semi-quantitative food frequency questionnaire
TMAO	Trimethylamine *N*-oxide
TNF-α	Tumor necrosis factor-alpha
UPFs	Ultra-processed foods
WHO	World Health Organization
VLPDs	Very-low-protein diets
